# Professionalism in the dental practice: perspectives from members of the public, dentists and dental care professionals

**DOI:** 10.1038/s41415-022-3994-3

**Published:** 2022-04-22

**Authors:** Dorottya Cserző, Alison Bullock, Jonathan Cowpe, Sophie Bartlett

**Affiliations:** 41415105428001 CUREMeDE, School of Social Sciences, Cardiff University, UK; 41415105428002 School of Dentistry, Cardiff University, UK

## Abstract

**Introduction ** This paper examines views on professionalism in the dental practice workplace through a thematic analysis of data from eight focus groups.

**Methods ** Focus groups were conducted with 19 dentists, 13 dental care professionals and 19 members of the public in England and Wales. The research was part of a larger mixed-methods study of professionalism in dentistry commissioned by the General Dental Council.

**Results ** The four most prominent themes in the focus group data were: communication, the cost of treatment, the role of the dental team and consequences of professionalism concerns. Participants agreed that these are fundamental professionalism issues, although there was some difference of opinion about addressing them. There was disagreement about the responsibilities of different members of the dental team in maintaining professionalism.

**Conclusion** We conclude that communication skills training should be a central part of the professionalism at all levels of training. Education about team working could foster a more collaborative approach to professionalism across the dental team and support good, patient-centred oral healthcare. Support and guidance are required to help professionals reflect and learn from mistakes.

## Key points

- Highlights the importance of communication skills as a core element of professionalism education.
- Suggests that education about team working could foster a more collaborative approach to maintaining professionalism within the dental practice.
- Recognises that lapses in professionalism can occur and should be reflected upon as part of ongoing learning.

## Introduction

Professionalism is a complex concept, open to interpretation depending on the situation, the context and the actors in play. The General Dental Council's (GDC's) *Standards for the dental team*^1^ set out nine principles which relate to aspects of professionalism, both organisational (for example, having an effective complaints procedure and maintaining and protecting patients' information) and personal (such as putting patients' interests first, effective communication and personal behaviour that maintains patients' confidence). These are reflected in Zijlstra-Shaw *et al*.'s definition which refers to 'professional responsibility and accountability'.^2^ These authors identify as important self-awareness and awareness of others, both patients and other members of the dental team. Similarly, lack of insight, endangering the self or others and lack of respect were reported by Taylor and Grey as unprofessional behaviours.^3^ Wellie defined professionalism as 'the social contract between the profession and the public which entails a collective responsibility of the members of the profession to serve the public good'.^4^

Professionalism is important because how patients are treated, clinically and personally, affects the trust they have in their dental professionals and whether they return for continued care.^5^ Teaching professionalism is a complex task and requires a combination of approaches.^6^^,^^7^^,^^8^^,^^9^ Challenges arising from demand for cosmetic treatments and use of social media and how, in a digital age, behaviours outside the workplace may compromise trust in the professional, have added urgency to the need for ongoing review of what it means to act with professionalism and how perspectives differ.^10^^,^^11^^,^^12^^,^^13^ Certainly, arguments have been made for further study of patient views.^14^ This article adds to the literature by presenting the results of a thematic analysis of focus groups on the topic of professionalism, comparing perspectives from members of the public, dentists and dental care professionals (DCPs). Our aim is to identify and compare which elements are prioritised by each of the three groups and reflect on the implications of diverging perceptions, focusing on behaviour in the workplace. We conclude by exploring the implications of our findings for professionalism education in dentistry.

## Methods

The focus groups were part of a larger mixed-methods study of professionalism in dentistry commissioned by the GDC and carried out by a research team from the Association for Dental Education in Europe (ADEE), largely based in Cardiff University.^15^ The full report contributed to the GDC's ongoing work on promoting professionalism (https://www.gdc-uk.org/information-standards-guidance/standards-and-guidance/promoting-professionalism). Focus groups were conducted in late 2019 in three locations in England and Wales, consisting of dentists, DCPs and members of the public. The aim of the focus groups was to explore perceptions of professionalism across the different groups. The option to attend a focus group was advertised to dental professionals by Health Education England Midlands and East and Health Education and Improvement Wales. Participants from the general public were recruited by Community Research and the local Community Health Council in South Wales. Each participant was provided with an information sheet and written consent was obtained. The focus group discussion topics as reported in this paper are listed in Box 1. The focus groups were led by members of the research team and audio recorded. Audio recordings were transcribed verbatim and anonymised. Ethical approval for the study was granted by Cardiff University (SREC/3390). Participating dental professionals were offered two hours of certified continuing professional development (CPD). One author (DC) carried out an inductive thematic analysis^16^ of the transcripts using NVivo Pro 12. The process involved creating a detailed thematic map covering all transcripts. The analysis was refined through discussions with authors (AB, JC, SB) at regular project team meetings and consultations with an expert reference group consisting of dental professionals. Details of the full set of themes and their distribution across the focus groups can be found in the report.^15^ In this paper, we discuss four of the most prominent themes and consider the implications of the differences between the groups.

We ran eight focus groups: four dentist groups, three groups with members of the public (here on referred to as the public group) and one DCP group (see Table 1). We also held individual telephone interviews with two DCPs (dental nurses) who wished to take part but were unable to attend the DCP focus group. The dentist groups had a total of 19 participants, including those newly qualified, as well as established dentists with decades of experience. The DCP group had 13 participants. There were eight dental nurses, two hygienists/therapists and two dental technicians. At least three of the attendees were actively involved in education and management. The public groups had a total of 19 participants with an age range of 20-60+. Some reported attending dental practices as private patients and others as NHS patients.

**Table 1:** Focus group participants

Group	Participants	Men	Women
Dentist Group 1	5	4	1
Dentist Group 2	2	0	2
Dentist Group 3	9	4	5
Dentist Group 4	3	1	2
**Subtotal**	**19**	**9**	**10**
DCP Group	13	2	11
DCP telephone interviews	2	0	2
**Subtotal**	**13**	**2**	**11**
Public Group 1	7	2	5
Public Group 2	6	3	3
Public Group 3	6	3	3
**Subtotal**	**19**	**8**	**11**
**Total**	**51**	**19**	**32**

Box 1 Focus group topic guide (reproduced from the original GDC report)^15^

- What does professionalism mean to you?
- What things would you consider to be 'unprofessional'? What would cause patients to 'lose trust' in a member of the dental team?
- In terms of professionalism, what do you think matters most to the public? Do you think the public's view of what matters is the same or different to what dental professionals think?

## Results

Three main themes were identified as important aspects affecting how the professionalism of the dental professional was judged. Firstly, communication was highlighted as a core element of professionalism. The second theme, cost of treatment, was contrasted against the principle of putting patients' interests first. Thirdly, there were discussions about the role of different members of the dental team in creating and maintaining a professional atmosphere within the dental practice. In addition to these three themes, the focus groups also discussed the consequences of professionalism concerns. Although these were the most prominent topics across all the groups, there were differences in the focus and framing of the issues. We present the views of each group on these four topics.

### Communication

The majority of the discussion around communication in the public groups focused on describing 'good' communication practices. From their perspective, the most important aspect was that the dental team should 'put the patient at their ease', mitigating fear of treatment. Patients also wanted to be 'treated like a person' and kept informed:

- '*I've got a very good dentist because he's friendly and approachable and you can talk to him and the staff are the same*' (Public Group 1).

The most common complaints raised in the focus groups with members of the public concerned poor communication or that the manner was perceived as rude:

- '*There are occasions where they won't even look at you. There's no eye contact because they look down at the desk and they just talk*' (Public Group 3).

Dentists highlighted challenges in communication they encountered during their work. The main difficulty was giving patients the 'right amount' of information in a limited time frame; too much detail could deter patients from having necessary treatment. There were also complaints about 'difficult patients':

- '*You've got to find out how much information the patient would want and give them the right amount of information. I've had situations where anxious patients then have not gone through with the procedure when they've been given all the information*' (Dentist Group 1).

In terms of poor communication, dentists identified similar issues to the public: that it is deficient, or rude in tone. There was disagreement as to whether communication skills can be taught; some argued that such skills are innate, others that they can be developed through training and experience.

The importance of good communication was also stressed in the DCP focus group and DCP telephone interviews. When providing definitions of professionalism, communication was a core element. In some cases, DCPs suggested patients care more about communication than clinical competence. Their view of good and poor communication in the surgery very much aligned with the other groups:

- '*I think there's plenty of very, very, very good clinicians out there who are very clinically competent that may lack in areas such as communication. And unfortunately we know that the first impressions sometimes leaves a lasting impression*' (DCP telephone interview).

### Cost of treatment

Members of the public raised concerns that some dentists may be 'profit-oriented' and prioritise financial gain over patient interest. Although no evidence was shared to support this claim, a lack of trust in the motivations of dentists was expressed in all three public focus groups:

- '*I would say that they focus strictly on the money and try to sell me things just for the sake of money, not because I need it*' (Public Group 2).

Dentists were aware of this public perception and some expressed frustration that they are seen as 'greedy' by the public:

- '*The public think that we're greedy, we charge too much, we drive fast cars, we live in lovely country houses*' (Dentist Group 3).

No dentist admitted to being 'profit-driven' but some noted that they 'have a small business to run' and need to balance income with outgoings on staff costs, material costs and other expenditure in the context of fulfilling (NHS) contracts. Others mentioned high-profile fraud scandals within the dental profession.

DCP participants who had experience of working in the hospital dental service as well as in the general dental service suggested that high street practices were more driven by the need to make a profit:

- '*I think there's still a difference between general practice and hospital because obviously general practice is fundamentally a business, isn't it? So it's always going to be driven by money unfortunately*' (DCP Group).

### The dental team

The focus group discussions highlighted that professionalism is expected not only of dentists but of all members of the dental team. All three public groups mentioned the role of the receptionist as the first point of contact. They also mentioned dental nurses and hygienists as members of the team who may talk to the patient more than dentists in some cases:

- '*My experience is the dentist doesn't come out to greet you. It's the dental nurse that will come out and do that*' (Public Group 3).

Dentists had more extended discussions about different roles in the dental team than those in the focus groups for members of the public. There were some dentists who highlighted differences in responsibility and others who stressed shared responsibility and working together as a team:

- '*The buck always stops with the dentist, doesn't it? So if there is an incident, usually we are held more accountable for that incident*' (Dentist Group 4)
- '*I don't think that I could give the care I give unless I had a dental nurse who was actually preparing all the equipment [...] to me it's a team [...] and I would expect my dental nurse to tell me if something wasn't right*' (Dentist Group 2).

DCP participants highlighted that the behaviour of each member of the dental team reflects on the team as a whole and expressed enthusiasm about the increased remit of dental nurses. They also noted that as an employee lower in the professional hierarchy of the practice, it would be difficult to challenge problematic practices:

- '*I think as a nurse, if the dentist is a bit abrupt, we over-compensate [...] and generally, then the dentist is okay because the nurse made such an effort to welcome the patient. Calm the patient down. It gets reflected as a team then*' (DCP Group).

### Consequences of professionalism concerns

None of the public participants reported making a complaint about a perceived lapse in professionalism. However, members of the public in all three groups gave examples of changing dentists when they were unhappy with their experience. Although we note that some of the examples may not have reflected a lapse in clinical professionalism (such as an unsuccessful treatment), examples did include communication problems.

Dentists talked about 'defensive dentistry' and a 'climate of fear'. DCPs provided further examples of dentists who are 'terrified of being sued'. In two of the dentist groups, participants criticised the GDC complaints process, expressing a perception of the dentist being 'guilty until proven innocent' of a professionalism concern. Some dentists also shared narratives illustrating the severe impact of being investigated and the time it takes to investigate a case, even if the outcome is positive, which could have a profound and long-term impact on the dental professional:

- '*When somebody does have a complaint, people would just crumble and never want to go back to work again, but yet they're expected to carry on the next day and the next day and the next day, until it's dealt with*' (Dentist Group 1).

## Discussion

In discussing these findings, we note that our participants are not intended to be a representative sample of the public, dentists, or DCPs. Rather, we highlight the issues which arose as important in the course of the discussions with our three groups of participants and draw comparisons where appropriate.

All three groups agreed that communication between the patient and the dental team is one of the most important factors in determining the quality of a dental encounter. The importance of effective communication is reflected in the GDC standards in their second principle. There was agreement that good communication puts patients at ease and makes them feel respected. When the encounter is poor, it is often due to communication limitations (for example, not giving enough information to the patient or behaving in an abrupt manner). There is ample evidence that communication is a skill that can be developed with the right training.^17^^,^^18^^,^^19^^,^^20^^,^^21^ By framing it as an innate ability, professionals close themselves to developing a skill which has been identified as a key factor in formal complaints.^1^

The perception of the 'greedy dentist' driven by profit rather than patient care need appeared in the public and dentist focus group discussions. The public provided no evidence to support this picture and dentists expressed their frustration about being represented in this light. Their discussions highlighted the need to balance competing demands: patient need, material costs, staff costs and time pressures dictated by contracts. DCPs in the focus groups who had experience of both hospital and general dental practice settings recognised that the latter was necessarily concerned with profit. If patient perceptions of the 'greedy dentist' were accurate and some dentists determined treatment on the basis of financial gain rather than patient need, that approach would be in direct contradiction to the GDC's first principle^1^ and thus represent unprofessional behaviour.

In discussing different roles in the practice, the public referred to the receptionist several times. This is notable because receptionists were not mentioned in the dentist and DCP groups. The dentist groups were divided between those taking a more collaborative approach and those emphasising the hierarchy of roles. In the DCP groups, participants welcomed the increased remit of dental nurses and expressed a desire for DCPs to ensure high standards of professionalism.

Some of the dentist participants painted a stark picture of professionals being terrified of being sued. They stressed the severe consequences of complaints procedures, regardless of the outcome. Having a clear and effective complaints procedure is also a requirement from the GDC (principle 5). In contrast, discussion in the public focus groups suggested that patients are more likely to leave a dental practice than make a formal complaint. Complaints were discussed in a hypothetical sense but there were no examples shared of complaints made against dentists. However, several members of the public shared narratives featuring leaving a practice. The difference in the way the groups discussed dealing with professionalism concerns suggests that although complaints are rare, the consequences can be serious.

Lapses in professionalism are context-dependent and therefore difficult to define. We argue that distinction needs to be drawn between one-off mistakes and behaviours warranting formal complaint and investigation by the regulatory body. Complaints about professionalism can be detrimental to the individual's mental health and can affect service provision. A one-off mistake, something out of character and without implications for patient care, should be an experience the individual is able to learn from to avoid it being repeated. In the case of a one-off mistake, rather than a pattern of behaviour, there is scope to update current guidance and provide ongoing optional support for dental professionals.

## Conclusion

From our inductive thematic analysis, we gained an in-depth understanding of the perceptions of our participants. Our sample is by no means representative of the whole population but the research nevertheless highlights issues that currently shape relationships between dentists and DCPs and the public and the dental profession. In these discussions about professionalism, compared with members of the dental team, views expressed by members of the public differed, particularly in relation to communicating with patients: the role of receptionists, the main point of contact with the public, were given notable consideration in the public focus groups. The contribution of the whole dental team (not only DCPs and dentists) is worthy of consideration in educational activity. Our research highlights the importance of training in communication skills as a core element of professional training for all members of the dental team. There is scope to investigate the role of dental receptionists, particularly in relation to communicating the cost of treatment to patients and further explore the views of DCPs, who have so far received less attention than those of dentists.

Within the undergraduate curriculum, the domain of professionalism is closely intertwined with communication, as well as leadership and this is reflected in our findings. Although the focus of the research was professionalism, clear implications are raised for learning and teaching about communication skills, teamwork and leadership. We acknowledge the importance of laying the foundations of good communication skills, teamwork and interprofessional collaboration in undergraduate education and acknowledge the need to build on these skills through continuing professional development.

In this article we have limited our focus to behaviours in the dental practice; it is also necessary to consider behaviours outside the workplace and the sometimes blurred boundaries between behaviours in and outside the workplace.We end with the plea that professionalism is not seen as a complete absence of errors, but rather the ability to reflect upon and learn from mistakes.
